# Anesthesiology Safety in Morocco: A Synthesis of Three Studies Towards Integrated Multidisciplinary Collaboration

**DOI:** 10.7759/cureus.89508

**Published:** 2025-08-06

**Authors:** Wafaa Harfaoui, Mustapha Alilou, Ahmed Rhassane El Adib, Mehdi Lekehal, Ayoub Bounsir, Lahcen Belyamani, Majdouline Obtel

**Affiliations:** 1 Epidemiology and Public Health, Laboratory of Community Health, Preventive Medicine and Hygiene, Faculty of Medicine and Pharmacy, Mohammed V University, Rabat, MAR; 2 Epidemiology and Public Health, Laboratory of Biostatistics, Clinical Research and Epidemiology, Faculty of Medicine and Pharmacy, Mohammed V University, Rabat, MAR; 3 Intensive Care Unit, Hospital Ibn Sina, Rabat, MAR; 4 Anesthesiology, Critical Care and Emergency Medicine, Faculty of Medicine and Pharmacy, Cadi Ayyad University, Marrakesh, MAR; 5 Laboratory of Simulation Research, Faculty Mohammed VI of Medicine, Mohammed VI University of Health and Sciences, Casablanca, MAR; 6 Department of Vascular Surgery, Hospital Ibn Sina, Rabat, MAR; 7 Pedagogical and Research Unit of Vascular Surgery, Faculty of Medicine and Pharmacy, Mohammed V University, Rabat, MAR; 8 Mohammed VI Foundation of Health Science, Mohammed VI University of Health and Sciences, Rabat, MAR; 9 Royal Medical Clinic, Mohammed V Military Hospital, Rabat, MAR; 10 Pedagogical and Research Unit of Emergency Medicine, Faculty of Medicine and Pharmacy, Mohammed V University, Rabat, MAR

**Keywords:** anesthesiology in morocco, challenges, health system reform, multidisciplinary collaboration, patient safety

## Abstract

Anesthesiology is crucial to modern medical care. In Morocco, significant progress has been made in anesthesiology patient safety since the early 20th century, thanks to advances in pharmacology, technology, and training. However, challenges persist that call for rigorous action.

Our study assesses the current state of anesthetic practice in Morocco and aims to propose an integrated framework adapted to the Moroccan context in order to sustainably improve anesthetic practice. Additionally, this article presents a systematic and structured review of the literature, accompanied by a contextual synthesis based on an adapted PRISMA method. The analysis is based on three main studies, chosen for their relevance and methodological quality, as well as on complementary references from databases.

Thus, the synthesis of the three selected articles provides a comprehensive view of anesthesiology safety issues. It describes international advances and promising innovations, as well as the challenges faced by low- and middle-income countries. In the context of Morocco, the findings highlight both convergences and divergences in patient safety practices. Specific challenges include territorial disparities, a shortage of professionals, financial and material constraints, insufficient continuing education, and the absence of an adequate legislative framework.

Additionally, this study highlights the initiatives underway and the implementations carried out by the new reforms, which aims to remedy persistent deficits and raise all aspects and areas necessary to adopt solutions appropriate to Moroccan realities. Finally, to enhance patient safety in Moroccan anesthesiology, a multidisciplinary collaboration is needed that prioritizes the ongoing development of national standardized guidelines, competency-based training with continuous maintenance of competence, and the establishment of clear legal and regulatory standards for licensing, accreditation, and minimum safety requirements during anesthesia. This strategy should integrate technological innovations tailored to local resources and socioeconomic realities, supported by continuous research and structured patient engagement. By addressing ethical, regulatory, and resource-specific barriers, these measures will help establish safe, high-quality anesthesiology in care delivery.

## Introduction and background

Anesthesiology is a fundamental pillar of modern medical care, being indispensable for performing complex surgical procedures while ensuring patient safety and comfort. Over the decades, this specialty has evolved significantly, transforming a practice once considered risky into a highly safe discipline. This transformation is a result of the introduction of new anesthetic molecules, advanced administration techniques, and sophisticated monitoring equipment [1]. In addition, the creation of learned societies has led to the establishment of rigorous protocols [2], helping to reduce mortality rates associated with anesthesiology. However, despite these major international advances, major challenges remain, with risks affecting both patients and healthcare professionals. In addition, socio-economic and organizational specificities represent a particular obstacle to the implementation of anesthetic safety in line with global standards, especially in low- and middle-income countries such as Morocco [3]. It is therefore essential to analyze these current challenges to propose concrete solutions that guarantee safe and effective anesthetic care.

Refocusing on the Moroccan context, it is important to emphasize that anesthesiology underwent significant changes, particularly during the Second World War, when using chloroform and ether, although common, was risky. In 1942, when the Allies landed in North Africa, the French African Army trained doctors in safer techniques, such as oxygen and nitrous oxide anesthesia [4]. Indeed, before the protectorate, anesthesiology assistants were trained in religious schools, but the first nursing school opened in Casablanca in 1941, reserved only for young French women residing in Morocco. After independence in 1956, Morocco suffered from a shortage of medical staff, leading to accelerated training and the creation of the first nurse anesthesiologist school in 1960 [5]. In addition, pioneers such as Dr. Victor Blanc and Dr. Philippe Garcia introduced contemporary practices, and in 1975, Prof. Slaoui became the first Moroccan physician to head the anesthesiology department at Avicenne Hospital, playing a key role in the evolution of the specialty [6]. Furthermore, the creation of the Moroccan Society of Anesthesiology and Intensive Care in 1984 helped to improve anesthetic practices and issue several recommendations on anesthesiology safety [7], and in 2015, in Berlin, Morocco signed up to the Helsinki Declaration on Patient Safety in Anesthesiology, demonstrating its commitment to the continuous improvement of anesthetic practices [8].

Despite the progress made, Morocco continues to face major challenges such as territorial inequalities in access to anesthetic care, mainly due to the concentration of health establishments in densely populated areas. Although the healthcare budget has recently seen a slight increase, it remains limited to 6-7% of the state budget, which is still insufficient to meet the sector's needs. This situation is exacerbated by an alarming shortage of anesthesiologists, most of whom are concentrated in the major cities, making access to specialist care even more difficult in less-favored regions. The inadequacy of the regulatory framework to the realities of the field, combined with insufficient funding for continuing education [3], underlines the need for urgent reforms to improve the quality and safety of anesthesiology care at the national level. Consequently, even if anesthesiology in Morocco has evolved significantly, it is crucial to continue efforts to comply with international standards and achieve the highest recommendations. In this context, our article aims to assess the current state of anesthetic practice in Morocco and to identify the strengths, shortcomings, and levers for improvement needed to optimize the quality and safety of anesthetic care.

To this end, we have selected three recently published articles from 2024, chosen for their relevance, complementarity, and topicality, which approach patient safety in anesthesiology from different angles. The first article, titled "Patient Safety in Anesthesiology: Progress, Challenges, and Prospects" [1], offers a global perspective on the evolution of safety in anesthesiology, highlighting progress made and persistent challenges; it also highlights technological innovations, providing a general context and future trends that can be applied to Morocco. Next, the second article, "Compliance With World Health Organization (WHO)-World Federation of Societies of Anesthesiologists (WFAS) Standards for General Anesthesia at Ibn Sina University Hospital Center, Morocco" [2], focuses on practice evaluation, analyzing anesthetic practices at Ibn Sina hospital, the country's largest healthcare facility, and compares these practices to international standards set by WHO and WFSA, identifying strengths and gaps for improvement. Finally, the third article, entitled "The New Reform of the National Health System in Morocco: An Opportunity to Meet the Challenges and Improve the Practice of Anesthesiology" [3], examines the potential impact of the Moroccan health system reform on anesthetic practice, identifying the specific challenges to be met and the opportunities for improvement it could offer. 

In addition to an in-depth analysis of its three founding articles, an expanded corpus of several sources was selected using the PRISMA 2020 guidelines (Preferred Reporting Items for Systematic Reviews and Meta-Analyses) [9], enabling us to identify optimization levers while contextualizing persistent challenges. Research was also conducted in electronic databases. Furthermore, the search strategy consisted of a systematic combination of keywords and phrases related to anesthesiology safety, covering all its issues and aspects. We aimed to achieve sustainable anesthetic safety by adopting a multidisciplinary collaborative approach that integrates not only the expertise of anesthesiologists and healthcare professionals but also that of biomedical engineers for technological innovation, legal experts for regulatory matters, psychologists for stress management and communication, and patient engagement for personalized care, as well as the involvement of managers and political decision-makers to optimize resources and develop appropriate health policies. What's more, this synergy between different areas of expertise is essential to meet the complex challenges of modern anesthesiology and ensure a comprehensive and sustainable improvement in anesthetic practice in Morocco.

## Review

Methodology

Study Design

A comprehensive analysis was carried out for this study, based on a rigorous systematic review of the literature, supplemented by a contextual synthesis. The main objective was to assess patient safety in anesthesiology in Morocco, based on three fundamental studies published in 2024 [1-3]. These were carried out as part of the doctoral thesis of the first author of these articles, conducted within the Doctoral Studies Center for Life and Health Sciences of the Faculty of Medicine and Pharmacy of Rabat, which is a part of a structured research program focusing on the challenges and prospects of patient safety in anesthesiology in Morocco. In addition, these complementary studies, carried out under the supervision of several professors and in collaboration with various co-authors, see their contributions duly recognized. Furthermore, an extensive contextual synthesis, based on a total of 68 references, was carried out using a thematic approach, and the extracted data were organized and analyzed around several key axes, including clinical, technical, organizational, regulatory, psychological, and educational aspects related to patient safety in anesthesiology. This thematic approach fostered a thorough and nuanced understanding of the subject, highlighting the various dimensions and interactions between factors. A critical and synthetic reading of sources was used to integrate a variety of perspectives, while preserving the coherence and scientific rigor of the synthesis.

Data Sources and Research Strategy

The research strategy adopted for this study is based on a systematic and rigorous exploration of the main scientific databases, enabling the selection of three fundamental studies published in 2024, chosen according to strict criteria guaranteeing their relevance, the quality of the analyses, the presence of co-authors, as well as the thematic relevance and topicality of the data in a context marked by the scarcity of local empirical studies in Moroccan anesthesiology. This work, combining quantitative and qualitative methodologies validated by peers, covers a triadic thematic spectrum totalling 254 references, which are distributed as follows: patient safety in anesthesiology, between progress, challenges and perspectives (143 references); compliance with WHO-WFSA standards in Morocco's largest healthcare establishment (26 references); and the recent reform of the Moroccan healthcare system and its impact on anesthesiology (85 references).

The integrated bibliographic wealth underlines the importance of selecting relevant studies while avoiding redundancy in an emerging field of research. Although published in 2024, these studies are based on distinct collection periods, ranging from 2019 to 2024 for the exhaustive review of the first article on patient safety in anesthesiology, and five months in 2021 for the second article on Compliance with (WHO) and (WFAS) standards, and between 2021 and 2024 for the third article analyzing the impact of the recent reform on anesthetic practice.

A second approach with an enriched contextual synthesis involved carrying out an in-depth systematic review to analyze patient safety in anesthesiology in the international context, particularly in Morocco, from different aspects, in order to deepen understanding and analysis of the subject. In line with PRISMA (Preferred Reporting Items for Systematic Reviews and Meta-Analyses) recommendations [9], this review was conducted with rigor and transparency, and searches were also carried out in electronic databases such as Web of Science, PubMed, Scopus, and Google Scholar. 

The search strategy was based on a systematic combination of keywords and expressions covering the full range of anesthetic safety issues, including: patient safety in anesthesiology; anesthesia practices in Morocco; evolution of anesthesia practices; multidisciplinary collaboration; biomedical innovations in anesthesiology; technological advances in anesthesiology; regulations and legislation in anesthesiology; ethics and standards in anesthesiology; psychological impact on patients and professionals; economic stakes of anesthetic health; participation and role of the patient in anesthesiology; research in anesthesiology; continuing education of anesthesiologists. Thus, these main terms were systematically combined using logical operators, enabling the concepts to be precisely crossed or the search to be extended with synonyms.

Thanks to this approach, it was possible to simultaneously optimize the relevance and exhaustiveness of the results, facilitating the rigorous selection of documents integrating several thematic dimensions, as well as the inclusion of synonyms or closely related expressions. Subsequently, the studies were then selected and evaluated according to a methodical and rigorous approach, with an initial screening of titles and abstracts to identify relevant publications, followed by an in-depth review of the full texts to assess in detail the eligibility of each selected article.

Inclusion and Exclusion Criteria

Inclusion criteria covered issues such as anesthetic safety, training, technological innovations, legal/psychological aspects, and policy. In addition, the search was limited to articles published in French and English, the publication period having been deliberately extended to enable a comparative analysis of developments in the field. In addition, the selection was based on various types of sources, including literature reviews, systematic reviews, meta-analyses, original research articles, books and book chapters, and government documents (national/international). Conversely, all documents outside the field of anesthesiology, publications in languages other than French or English, incomplete conference abstracts, and duplicates were excluded from the analysis.

Study Selection and Data Extraction

The studies were selected by two independent reviewers on the basis of predefined criteria covering methodological quality, relevance, and contribution to the research objectives, including three primary studies from the first author's doctoral project. The selection was carried out in two stages: an initial screening of titles and abstracts was used to identify publications likely to meet the objectives, followed by a full reading of the selected articles to determine their eligibility. The bibliographic references were then managed using Zotero software, and the data extracted was then organized according to a thematic approach, enabling analysis of the clinical, organizational, regulatory, psychological, and educational dimensions relating to patient safety in anesthesiology. This targeted approach resulted in an in-depth, coherent synthesis of the findings, in line with international recommendations calling for a detailed analysis of key studies relevant to the research question.

Quality Assessment

The methodological quality of the included studies was assessed using a grid designed specifically on the basis of fundamental criteria widely recognized in scientific methodology. The grid focused on five key issues essential to the validity and relevance of the studies: clarity of research objectives, appropriateness of methodology, identification and consideration of potential biases, reliability of results, and relevance of conclusions. As a result, this approach led to a rigorous and systematic analysis of all the documents and articles included, demonstrating in-depth critical vigilance. Moreover, given the diversity of the types of studies examined, the use of a single standardized tool might have proved insufficient to capture all the specific methodological features; thus, adapting the grid ensured a consistent, transparent, and reproducible assessment. Indeed, this approach ensured that only data of high methodological quality were included in the synthesis, reinforcing the reliability and validity of the results and contributing to a thorough and balanced understanding of anesthesiology safety issues at the global and national levels.

Results

First Approach

This article presents the results of three studies that explore common themes related to patient safety in anesthesiology. Moreover, this diversity highlights both points of convergence and divergence in patient safety and offers an enriching perspective on advances and challenges in the field of anesthesiology, both globally and in Morocco.

In analyzing the first article, entitled ”Patient Safety in Anesthesiology: Progress, Challenges, and Prospects”, it traces the evolution of anesthesiology from empirical practices to modern technological innovations, highlights notable advances in patient safety while underscoring persistent challenges, especially in low- and middle-income countries, and offers promising perspectives on emerging technologies that raise important ethical and technical questions. Table 1 provides a summary of the key points mentioned in the article [1].

**Table 1:** Summary of the Main Advances, Challenges, and Innovations Discussed in “Patient Safety in Anesthesiology: Progress, Challenges, and Prospects”

Categories	Details
Main advances	Ancient origins: (1) Use of natural substances and mechanical techniques. (2) 19th century: Major discoveries (ether, chloroform, nitrous oxide). (3) 20th century: Introduction of halogenated anesthetics, Development of intravenous anesthetics, Improvement of administration and monitoring techniques, development of specialized equipment and creation of professional associations.
Key current challenges	(1) Risks associated with the anesthetic procedure: Side effects and complications of drugs, technical problems (difficult intubation, equipment malfunctions), and human error. (2) Patient-related risks: Comorbidities, drug interactions, genetic variability. (3) Constraints on professionals: Stress, high workload, risk of legal action. (4) Challenges in low-income countries: Lack of resources and infrastructure, staff shortages, limited access to safe and affordable anesthesiology care.
Technological Innovations	(1) Simulation and virtual reality: advanced training for anesthesiologists, and Improving technical and non-technical skills. (2) Artificial intelligence: Clinical decision support, prediction of complications and optimization of anesthetic administration. (3) Genomics: Personalizing treatments and predicting individual risks. (4) Robotics: Assistance with certain tasks (intubation, locoregional anesthesia) and development of remote anesthesia.
Potential benefits	Improved safety and efficiency of anesthesiology care based on personalized treatment, ongoing professional training, and optimized resource management. The result will be greater access to quality care for patients.
Limits and ethical considerations	The technical challenges of AI in anesthesiology include a lack of quality data, algorithmic biases, and ethical concerns about data confidentiality and liability in the event of error. Socio-economic issues, unequal access, and high implementation costs also pose shortcomings, while the lack of a regulatory framework necessitates further rigorous studies to validate efficacy and safety.

In referring to the second article 'Compliance With World Health Organization (WHO)-World Federation of Societies of Anesthesiologists (WFAS) Standards for General Anesthesia at Ibn Sina University Hospital Center, Morocco', it examines compliance with WHO-WFSA international anesthesiology standards at the Centre Hospitalier Universitaire Ibn Sina in Rabat( the largest health facility in Morocco, In analyzing the various aspects, generally satisfactory compliance is noted, although there is still room for improvement, particularly about continuing education, neuromuscular monitoring and the installation of post-interventional monitoring rooms. Table 2 provides an overview of the main points discussed in the article [2].

**Table 2:** Summary of the Various Aspects, Opportunities, and Shortcomings Discussed in “Compliance With WHO– WFSA Standards for General Anesthesia at Ibn Sina University Hospital Center, Morocco” ECG: electrocardiogram; BP: blood pressure; SpO2: blood oxygen saturation; PONV: postoperative nausea and vomiting; WHO-WFSA: World Health Organization-World Federation of Societies of Anaesthesiologists

Aspect	Key results
Patient characteristics	37.2% aged 36-55, 52% women, 48% men, 67.2% classified ASA I
Professional aspects	98% of anesthesia procedures performed by anesthesiologists, 55.2% of nurse anesthetists with occasional continuing education, 94.4% report good team spirit
Pre-anesthetic practices	65.6% pre-anesthetic consultations, 89.6% pre-anesthetic visits, 89.6% use of checklist, 73.8% premedication
Anesthesia techniques	85.2% tracheal intubation, 7.6% face mask, 7.2% laryngeal mask, Equipment for difficult intubation available in all hospitals
Anesthetic agents	92% propofol, 92% fentanyl, 82% rocuronium, 75,2% sévoflurane
Monitoring	100% standard monitoring (ECG, BP, SpO2), 94% capnography
Intraoperative management	91.2% crystalloid filling, 18% transfusion strategies, 41.6% intraoperative complications (17.2% cardiovascular)
Post-operative management	80% postoperative analgesia, 58.8% presence of an ICU, 18% transfers to intensive care, 12% postoperative complications (mainly PONV)
Compliance with WHO-WFSA standards	(1) Good overall compliance, but room for improvement. (2) Gaps: continuing education, neuromuscular monitoring, ICU

In examining the third article, 'The New Reform of the National Health System in Morocco: An Opportunity to Meet the Challenges and Improve the Practice of Anesthesiology', dedicated to the reform of the Moroccan health system, it aims to transform the medical sector in depth, particularly in the field of anesthesiology. It defines the essential pillars of this reform while raising major national challenges and proposing ambitious measures and necessary interventions to guarantee equitable, safe, and quality access to anesthesiology care for all Moroccan citizens, in line with international recommendations. Table 3 summarizes the main points raised in the article [3].

**Table 3:** Summary of the Challenges, Opportunities, and Measures Identified in “The New Reform of the National Health System in Morocco: An Opportunity to Meet the Challenges and Improve the Practice of Anesthesiology”

Aspect	Details
Context of the reform	Since 1959, Morocco's healthcare system has evolved significantly, marked by several reforms and laws. In 2021, the country adopted framework law 09.21 on social protection, aimed at strengthening the healthcare system and guaranteeing equitable access to care. This development continued in 2022 with the enactment of Framework Law 06.22, which establishes the foundations of the national healthcare system. The main aim of these reforms is to improve the accessibility, quality, and safety of healthcare for all Moroccan citizens.
Key aspects of the new reform	Morocco is committed to universal medical coverage by 2025, while promoting good governance through the creation of several bodies, such as the High Health Authority, health groups, the Moroccan Agency for Medicines and Health Products, and the Moroccan Agency for Blood and Blood Derivatives. At the same time, the country is emphasizing the development of human resources and the upgrading of healthcare provision to improve the quality of healthcare services. The digitalization of the healthcare system is also a priority, aimed at modernizing infrastructures and facilitating access to healthcare for all citizens.
Challenges of anesthetic practice	Morocco faces challenges such as territorial disparities and inter-regional inequalities, particularly between regions and between urban and rural areas. In addition, the lack of financial resources is a cause for concern, with only 6-7% of the state budget allocated to health, whereas the WHO recommends allocating 12%. The shortage of anesthesiologists is alarming, with just 658 physician anesthesiologists currently practicing across the country, including 196 in the public sector and 462 in the private sector, whereas the WHO sets a target of 20 anesthesiologists per 100,000 inhabitants. In addition, continuing training for healthcare professionals is inadequate, receiving less than 2% of the budget allocated to training. Finally, the current legal and administrative framework remains inadequate, particularly for the practice of nurse anesthesiologists, thus limiting the efficiency of the healthcare system.
Measures taken and impact on anesthesiology	Morocco has undertaken significant efforts to strengthen its healthcare system, illustrated by an increase in the healthcare budget, which will reach 30.7 billion dirhams in 2024, compared with just 6.1 billion in 2006. At the same time, the country has improved its infrastructure, with 590 rehabilitation projects and 369 construction projects completed between 2017 and 2023. In terms of recruitment, 42,700 budgetary positions have been created between 2017 and 2024, while an ambitious target has been set to double the number of doctors and triple that of nurses by 2030 ,also the digitization of the sector is underway, with the implementation of an integrated health information system. Finally, the adoption of laws governing the healthcare professions is helping to structure and improve the quality of the services offered.
Outlook for the ministry	Morocco aims to achieve universal health coverage by 2025, a key objective for guaranteeing equitable access to healthcare for all citizens. To this end, the country is committed to training 90,000 healthcare professionals by the same deadline, establishing a ratio of 24 professionals per 10,000 inhabitants. To combat territorial inequalities, hospital construction projects are planned in under-resourced regions. In addition, efforts will be made to improve the quality and safety of care, notably through the accreditation of healthcare establishments. Finally, strengthening governance is a priority, illustrated by the creation of public bodies dedicated to the management and supervision of the healthcare system.
Effective implementation of measures in the face of persistent challenges	The implementation of the new health system reform in Morocco is based on several major axes, aimed at overcoming current challenges. Indeed, to guarantee the effectiveness of these reforms, in-depth research is essential to monitor their evolution. Longitudinal studies and regular evaluations will enable progress to be measured, obstacles to be identified, and strategies adjusted accordingly. This approach is essential to improve not only the healthcare system as a whole but also the practice of anesthesiology in Morocco.

Second Approach

**Figure 1:**
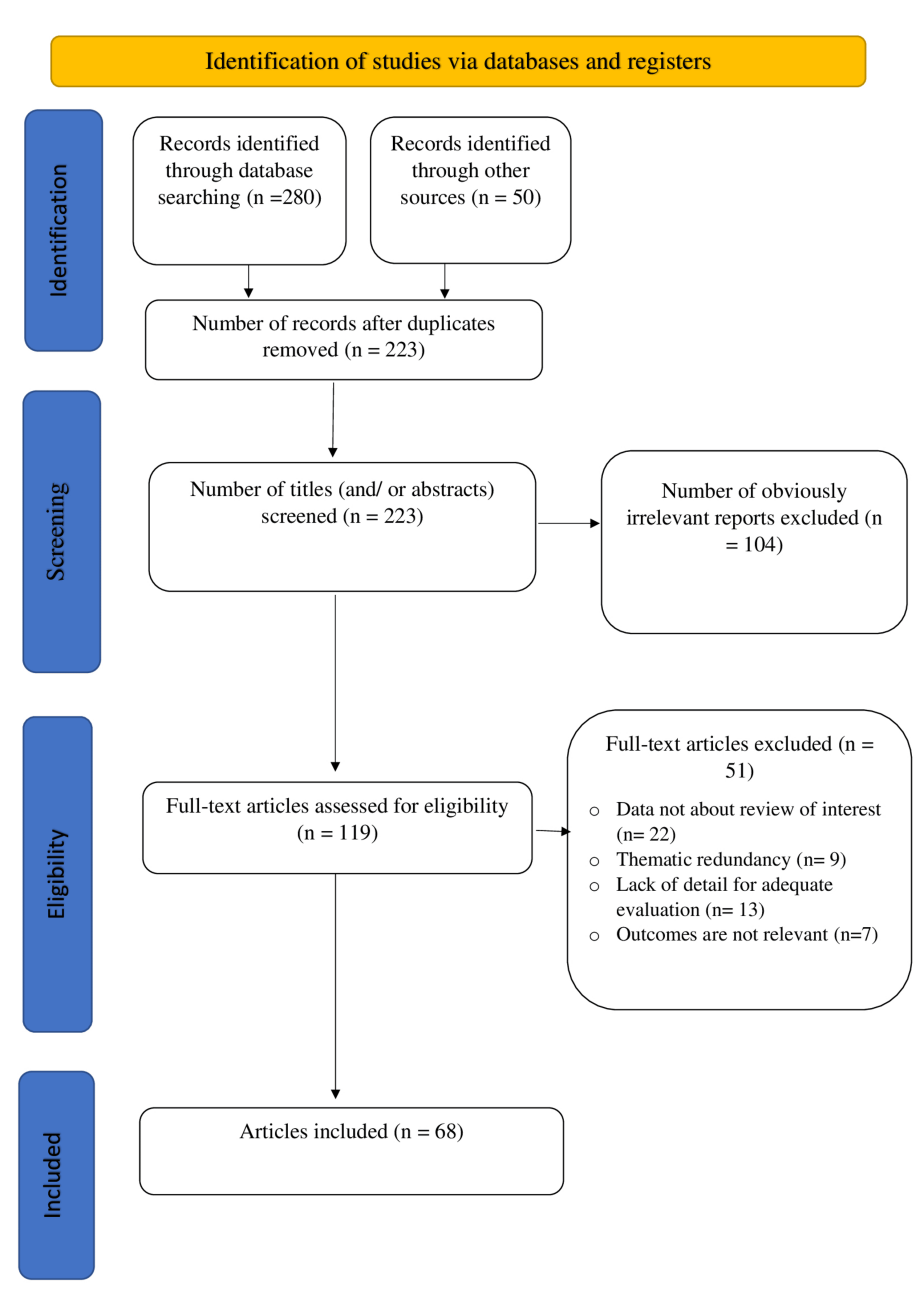
PRISMA flow chart illustrating the study selection process

Study selection process: The study selection process, illustrated in Figure 1 according to the PRISMA 2020 recommendations, took place in four distinct phases. However, during the identification phase, a total of 330 references were identified, including 280 from electronic databases (PubMed, Web of Science, Scopus, Google Scholar) and 50 additional references identified by other sources, including manual searches of reference lists of relevant articles, including the three selected studies.

Following this initial identification, the selection process involved two main stages. Firstly, deduplication eliminated redundant references, reducing the corpus to 223 unique documents. Secondly, the preliminary selection consisted of a careful examination of titles and abstracts, which led to the exclusion of 104 documents that were clearly irrelevant according to the defined criteria. In addition, only documents written in French or English were retained, while those written in other languages were discarded.

During the eligibility phase,119 full-text articles were subjected to rigorous evaluation. In addition, this analysis led to the exclusion of 51 articles for a variety of precisely documented reasons, including data not relevant to the review of interest (n=22), thematic redundancies between articles (n=9), lack of detail for adequate evaluation (n=13), and results not relevant to the objective of the review (n=7).

The final phase of inclusion selected 68 studies, which constitute the corpus analyzed in this review.

Methodological analysis of included studies

The methodological analysis of the 68 references included in this synthesis reveals a generally satisfactory scientific quality. Indeed, the bibliographic corpus reflects this complementarity, combining scientific rigor and practical contextualization. More specifically, it includes original articles (clinical trials, observational studies, experimental research) that form the basis of empirical evidence, as well as narrative and systematic literature reviews that synthesize and update knowledge. In addition, normative and regulatory documents (decrees, codes, international standards) specify the legal and safety framework, as well as institutional communications and official reports, providing contextual and strategic national and international data. Finally, this panorama is rounded out by multimedia sources (videos, web presentations) and clinical resources (reference works, manuals, clinical guides), promoting the wide and continuous dissemination of knowledge through experience and training. 

The majority of studies have clearly defined objectives, a suitable methodology, and explicit consideration of potential biases, thus guaranteeing transparency and reliability. However, the robustness of the results varies according to the approaches employed: applied studies, notably prospective studies and randomized trials, provide reliable data with well-founded conclusions, while literature reviews and public policy analyses provide interesting perspectives, sometimes limited by a less rigorous protocol or the absence of experimental data. This methodological diversity thus ensures broad and in-depth coverage of anesthesiology safety issues, particularly in the Moroccan context. Ultimately, this wealth and variety of sources guarantee a solid, reliable, and multidimensional documentary base, conducive to a global analysis of advances, challenges, and prospects in the crucial field of anesthesiology safety.

Themes Covered

Analysis of the 68 selected references reveals six main macro-themes, each grouping a set of studies characterized by a single central argumentative pivot. This exclusive attribution guarantees the absence of double counting and ensures an exhaustive classification of articles. In addition, Table 4 below shows the distribution of references according to these themes, indicating for each category the number of references and the corresponding percentage of all included studies:

**Table 4:** Thematic distribution of analyzed references (n=68)

Themes	N	%
Historical and institutional aspects and information sources	11	16.2
Scientific and technological advances	13	19.1
Governance, healthcare policies, and standards	15	22.1
Legal, ethical and deontological frameworks	11	16.2
Psychological dimensions and patient-centricity	12	17.6
Training, professional development and quality	6	8.8
TOTAL	68	100

This presentation highlights the diversity and complementarity of the themes addressed in the scientific literature analyzed, reflecting the multiplicity of scientific, social, legal, and organizational issues relating to anesthesiology.

Discussion

Anesthetic safety is a major public health issue, fundamental to primary healthcare, and although its importance is widely recognized, its implementation differs considerably from country to country due to a variety of factors. Yet, in order to guarantee patient safety, it is imperative to mobilize appropriate and adequate resources to ensure equity and safety in this essential area. Indeed, the three studies reviewed, which reflect international and national research in anesthesiology, both worldwide and in Morocco, are distinguished by the richness of their content and the diversity of their approaches, offering a comprehensive overview of advances, challenges and future prospects in the field, while highlighting the importance of patient safety and improved anesthetic practices, while diverging on aspects such as technological innovation, institutional compliance, and systemic reforms related to governance and access.

International Level

Our article on patient safety in anesthesiology; progress, challenges and prospects, deals with the historical evolution and significant progress of anesthesiology, marked by a significant drop in mortality rates, from 1 per 2000-2500 in the 19th century to less than 1 per 200,000 today [1], showing that this improvement is due to the introduction of new anesthetic agents, advances in sophisticated administration and monitorings techniques, and the creation of professional associations. Indeed, pharmacology has shown its contribution to the development of safe and effective anesthetic agents. Today, pharmacogenetics and pharmacokinetics have made it possible to personalize treatments and predict individual risks [1]. Simultaneously, new research is focusing on anesthetics that have rapid, predictable, and easily reversible actions, aiming to improve safety and efficacy [10], such as N-arylpyrrole derivatives revealing potential without hemodynamic suppression [11]. Moreover, recent advances in neuroscience have clarified how anesthetics interact with the neural circuits of sleep and consciousness [12]; however, the complexity of these interactions calls for more clinical research [13].

The integration of biomedical engineering [14] has revolutionized anesthesiology equipment and monitoring, enabling the introduction of sophisticated devices such as advanced ventilation systems and reliable monitoring tools. At the same time, patient safety has been enhanced by the addition of alarm systems and precise monitoring methods. In addition, technological innovations such as simulation, artificial intelligence, and anesthetic robotics have revolutionized anesthetic practices, leading to clinical decision support, prediction of complications, optimization of anesthetic administration, as well as personalization of the care provided [1].

Though new technological advances bring benefits, they also pose ethical challenges [1], such as informed consent and confidentiality in anesthesiology [15]. Their integration also requires attention to human factors and regulations [16], and highlights the importance of involving lawyers to ensure compliance with ethical and legal standards, thereby protecting the rights of patients and professionals alike, and, it is crucial to involve political decision-makers in the definition of strategies, the establishment of regulatory frameworks and the development of public policies is a fundamental pillar in ensuring ethical and safe practices in anesthesiology. In addition, the creation of anesthesiology organizations and federations has put patient safety at the heart of their priorities, promoting guidelines and establishing a culture of safety within care teams. This has also fostered research, continuing education, and the advancement of knowledge, thus contributing to improved quality of care [1]. 

Despite significant advances, anesthesiology still presents significant risks associated with anesthetic procedures, such as side effects, complications, dosage errors, and technical problems. However, thanks to recent innovations combining biomedical engineering and artificial intelligence, robotics and genomics are helping to optimize clinical decision-making, anticipate complications, and personalize treatments according to patients' genetic characteristics [1]. Moreover, for patients whose complex comorbidities pose challenges for management, artificial intelligence can predict adverse events, detect complications early, and optimize diagnosis and treatment, so genomics personalizes anesthetic approaches to reduce risk [1]. Simultaneously, effective psychological interventions can significantly enrich patients' experience and support their recovery process during anesthesiology [17].

Also, patient engagement in the anesthesiology care process is paramount, as it promotes effective communication and helps reduce medical errors [18]. Risks associated with healthcare professionals, such as burnout, workload, fatigue, and human error, can be mitigated through robotics and artificial intelligence, which optimize medication management and decision-making, and through simulation, which helps to improve performance and technical and non-technical skills [1]. In anesthesiology, where patient safety and well-being are paramount, a thorough grasp of the laws and standards in force would reduce the number of lawsuits brought against anesthesiologists, who are often faced with complex ethical and legal dilemmas. Indeed, gaps in their knowledge can have serious consequences [19]. It is therefore imperative that legal experts ensure that these regulations are rigorously applied and that staff are trained in legal issues, thus helping to minimize liability risks. Their role is essential in managing litigation and optimizing clinical outcomes [20]. Then, to manage anesthesiologists' anxiety and stress, psychology is proving to be a key factor, drawing on coping mechanisms such as emotional support, mindfulness, relaxation techniques, and resilience training. These approaches contribute to their well-being and job satisfaction, while enhancing the quality of patient care [21].

In addition, it is essential to focus on quality training [22] and continuing professional development programs [23], as well as interprofessional training for anesthesiologists [24], in order to enhance the necessary interdisciplinary skills and prepare anesthesiologists to manage complex situations, avoid errors, and maintain high standards. A study shows that simulation-based interprofessional training improves the attitudes and teamwork of anesthesiologist residents and nurse anesthesiologist students, fosters a culture of collaboration, and empowers anesthesiology professionals through effective training methods [25]. On the other hand, in order to overcome persistent obstacles, the design of sustainable solutions is of prime importance, as low- and middle-income countries face considerable challenges in the field of anesthesiology [1], such as strengthening the anesthesiology workforce, ensuring fair remuneration, taking account of the local context, guaranteeing adequate training, and improving the availability of equipment and drugs, while investing in new technologies. Indeed, a multi-dimensional approach combining efforts at local, national, and international levels, as well as close collaboration between governments, health organizations, and international players, is essential to improve the practice of anesthesiology [1,26].

National Context

In Morocco, it is observed that anesthetic practice has undergone notable advancements over the years, as shown by three studies published in 1999 [27], 2010 [6], and 2021 [2]. However, despite their differences in methodology and sampling, key improvements have been observed.

Firstly, by increasing pre-anesthetic consultation from 47% in 1999 to 65.6% in 2021, with an intermediate increase to 57% in 2010, this trend reflects a growing awareness of the importance of pre-operative assessment to facilitate the identification of risks associated with anesthesia. In terms of anesthetic techniques, a major shift was observed in the use of anesthetic agents, noting that in 1999, halothane was used in 70% of cases, while by 2010, its use had decreased to 62%, with the introduction of isoflurane at 20% and sevoflurane at 11%. By 2021, halothane had completely disappeared, replaced by sevo, which accounted for 75.2% of cases. At the same time, thiopental, which dominated at 75% in 1999, has been largely replaced by propofol, used in 92% of cases in 2021, reflecting a move towards more modern, effective agents. Locoregional anesthesia has also gained in popularity, rising from 15% in 1999 to 32% in 2010. This increase shows a willingness to adopt adapted techniques for the benefit of patient comfort.

Secondly, laryngeal mask use, although marginal in 2010 at just 1%, has seen a slight increase, reaching 7.2% in 2021, indicating an improvement in clinical practices and a diversification of airway management techniques. With regard to patient monitoring, progress has been made, since in 1999, electrocardioscopic monitoring was performed in 65% of cases, while capnography was almost absent, then in 2010, monitoring was expanded with the addition of SpO2, reaching 90%, then in 2021, capnography was available in 94% of cases, and standard monitoring (ECG, BP, SpO2) was applied to 100% of patients. Finally, the introduction of anesthesia checklists in 89.6% of cases by 2021 demonstrates a reinforced commitment to patient safety.

In addition, perioperative care has been strengthened, with premedication increased from 28.5% in 1999 to 73.8% in 2021, followed by the creation of post-anesthesia care units (58.8%) and improved post-operative analgesia (80%) [2]. What's more, between 1999 and 2010, the number of anesthesiologists in Morocco rose considerably, from 300 to 524 [6], and now stands at a total of 658 [3], reflecting the growing need for anesthesiology care, the professionalization of anesthesia, and improved training for anesthesiologists. 

Indeed, the creation of the SMAAR society in 1984 played a key role in the structuring of anesthesia-intensive care in Morocco, notably with the organization of its first congress in 1985. Then, in the 2000s, it strengthened its regional influence through the Maghreb Federation and CARAF, a French-speaking pan-African club, supporting seven Maghreb congresses between 2000 and 2012. In addition, its commitment to patient safety has also materialized through national recommendations (2007), the Declaration of Helsinki (2015), and binding guidelines (2016). In 2023, a 20-step competency framework will standardize practitioner training. In response to the COVID-19 pandemic, the VigiCorona I-II (2020) programs have mobilized logistical and scientific resources. In addition, the organization of a number of hybrid events dealing with a variety of themes of crucial importance to all hospital practitioners [28]. In 2026, the 19ᵉ World Congress of Anesthesiologists in Marrakech will highlight these innovations, making SMAAR an exemplary model in the field [29]. All these developments testify to the progressive and remarkable progress affecting the various aspects of Moroccan anesthesiology care, highlighting relevant trends and improvements. However, our country, like all low- and middle-income countries, faces a number of problems and challenges in the field of anesthesiology safety, requiring appropriate, sustainable solutions.

Then, to evaluate anesthetic practice in Morocco and draw relevant conclusions, a comparison was made from the second article, on compliance with WHO and WFSA international anesthesiology standards, within the framework of the Centre Hospitalier Universitaire Ibn Sina in Rabat, the country's largest healthcare facility [2]. This analysis highlighted crucial elements. Among the strong points, the results reveal that the majority of anesthesia professionals adhere to international standards, as evidenced by the systematic presence of qualified and trained personnel, which has contributed to a significant improvement in practices. Indeed, the use of anesthesia checklists reflects a strong commitment to patient safety and to following established protocols, and preoperative visits and consultations are regularly carried out in line with international recommendations [30], with a finding of good understanding and cohesion within the team. This communication and mutual understanding are considered to be determining factors in the success of surgical interventions, in line with international recommendations. In addition, continuous patient monitoring protocols (ECG, non-invasive blood pressure, oxygen saturation, and capnography) are generally adhered to, which is vital to ensure patient safety during anesthesia. The hospital also boasts a full range of equipment for managing difficult intubations, enabling anesthesiologists to respond effectively to these situations and thus improve patient safety. Lastly, the use of appropriate anesthetic drugs, such as propofol and fentanyl, testifies to the adoption of modern anesthesiology practices, focused on efficiency and safety, while complying with international standards. Although the presence of post-anesthesia care units is only 58.8%, the administration of post-operative analgesia in 80% of cases shows a good level of post-operative care [2].

The survey highlights a number of worrying shortcomings. Indeed, 45% of nurse anesthesiologists do not benefit from regular continuing education, which can adversely affect the quality of care and services provided [2]. Coverage of the Post-Interventional Monitoring Room is insufficient, at just 58.8%, which compromises the safety of patients in the recovery phase and increases postoperative risk [31]. Moreover, the absence of neuromuscular monitoring increases the risk of complications, being crucial for adequate recovery after muscle block and to prevent residual weakness [32]. In this context, it is essential to establish clear protocols and conduct regular audits [33], while implementing a mandatory continuing education program, optimizing the SSPI in all OR departments and investing in specific equipment and monitoring [34], to improve anesthetic safety in the Moroccan context. 

The analysis of the article concerning the new reform of the national health system in Morocco highlights a significant opportunity to optimize anesthesiology practice and overcome challenges, notably the significant territorial disparities that limit access to anesthesiology care. In addition, the country suffers from a shortage of anesthetists, with only 658 anesthetist-resuscitators available, necessitating an urgent reinforcement of the workforce to reach a recommended ratio of at least 20 surgeons, anesthetists, and obstetricians per 100,000 inhabitants by 2030 [3]. Furthermore, financing is a significant weakness of the Moroccan healthcare system, with only 6-7% of the national budget dedicated to this sector. This is well below the 12% recommended by the World Health Organization (WHO), and less than 2% is also invested in continuing education; this situation severely limits the ability to invest in infrastructure, develop professional skills, and improve overall care [3]. Also, households bear 38% of health expenditure, despite the WHO recommendation that household contributions should not exceed 25% of total health expenditure, to avoid an excessive financial burden that limits access to care [35]. Moreover, Morocco ranks 94th worldwide (score: 46.79) in the Numbeo Healthcare Index for 2025 [36]; it is particularly worrying to note that the “Satisfaction with responsiveness in medical institutions” score only reaches 40.18 points. In addition, the assessment of medical staff skills is 49.51, while that of diagnostic equipment reaches 50.75 [37]. In addition, innovation is on the decline, as evidenced by Morocco's absence from the Bloomberg Innovation Index 2020 (up from 50th place in 2017) [38]. The country ranks 75th out of 131 in the Global Innovation Index 2020 [39], with low scores in research and development (25.9) and infrastructure (39.2). When it comes to preventive healthcare, Morocco ranks 132nd out of 142, with an alarming score of 10/100 according to the Hologic index [40]. In addition, all these data highlight the structural challenges hindering the modernization of specialized medical practices, including anesthetic practice, and call for rigorous measures to be taken. 

There is a lack of an appropriate legal framework for nurse anesthetists, which also hinders their professionalization, and highlights the need to adapt legislation to the local Moroccan context, in addition, in comparison with other international regulations, emphasizes a rigorous separation, such as in France, where the practice of anesthesiology is governed by several legislative texts, notably decree no. 94-1050 of December 5, 1994, relating to the technical operating conditions of health establishments about the practice of anesthesiology [41], and article R. 4311-12 of the French Public Health Code governs the skills and conditions of practice of state-qualified nurse anesthetists [42], while the Order of January 17, 2002 specifies the training required to obtain the state-qualified nurse anesthetist diploma [43], and the Code of Medical Ethics and the Civil Code require the informed consent of the patient, except in emergencies [44]. In the United States, however, anesthesiology is governed by a multi-level legal framework. Additionally, the Federal Code of Regulations Title 42, Section 482.52 on Condition of Participation for Anesthesia Services [45] requires that anesthesiology departments be well-organized, staffed by competent professionals, and that each patient receive comprehensive care, pre-, intra-, and postanesthesia, with rigorous documented follow-up, to guarantee the safety and quality of anesthesia care in hospitals. Secondly, since 2001, some states have been able to adopt an “opt-out” procedure, allowing them to free themselves from the obligation of medical supervision of Certified Registered Nurse Anesthetists (CRNAs) and grant them total autonomy. Furthermore, each state defines the scope of practice of CRNAs according to its own laws and regulations [46-47].

Indeed, in comparison with the countries mentioned, Morocco is characterized by highly divergent regulations and training of nurse anesthetists, which further widens the gap in terms of organization and medical practice. What's more, article 6 of law 43-13 requires the “effective presence” of the anesthesiologist during anesthetic procedures, without, however, clearly specifying the limits of technical delegation; this imprecision is all the more worrying as the country suffers from a significant shortage of anesthesiologists. As a result, the absence of a clear regulatory framework creates considerable legal uncertainty and ambiguity in everyday practice, compounded by the fact that the implementing decrees have still not been published since 2016 [3].

On the other hand, the implementation of ambitious measures is essential to overcome institutional and operational challenges, knowing that framework laws 09.21 on social protection and 06.22 on the national health system illustrate a political commitment to a more accessible and robust healthcare system. These include planned medical coverage and the creation of governance bodies, as well as the development of human resources, the rehabilitation of healthcare provision, and the digitization of the national healthcare system, which are crucial measures for improving the accessibility, quality, and safety of anesthesiology care. Indeed, collaboration between all stakeholders is therefore essential to overcome the challenges, and the implementation of the new reform will largely depend on their commitment and cooperation. Indeed, in-depth and rigorous long-term studies will need to be carried out to assess the direct impact of the reform on all aspects of anesthesiology [3].

Multidisciplinary Collaboration

Considering that safety is not a definitive state, but a process [48] that requires continuous adaptation to new innovations and lessons learned from past experience [49], and based on analyses highlighting the potential and shortcomings of patient safety in anesthesiology in Morocco, we find that interdisciplinary collaboration is crucial, and requires close coordination between different professionals, each bringing their own unique expertise. In addition, interprofessional communication between anesthesiologists, nurse anesthesiologists, surgeons, and other healthcare professionals is essential to have a significant impact on quality of care and patient safety [50]. On the one hand, continuing education for anesthesiologists significantly improves anesthesiology practice by fostering skills development, promoting patient safety, and integrating innovative teaching methodologies [51]. A survey of New Zealand anesthetists, with a response rate of 74%, reveals that interactive training, motivated by updating knowledge and accreditation, promotes concrete changes despite professional constraints [52].

Also, the expertise of biomedical engineers enables the development of advanced administration, monitoring, and equipment systems, which contribute significantly to improving anesthesiology safety through technological innovation and equipment maintenance [14]. According to a recent one-year study carried out in a tertiary care cancer hospital, 97.5% of identified malfunctions were quickly resolved thanks to proactive maintenance and effective intervention by the biomedical team. In a one-year survey of a tertiary care cancer hospital, 97.5% of identified malfunctions were quickly resolved thanks to proactive maintenance and effective intervention by the biomedical team, underlining the importance of these practices in ensuring patient safety [53].

Furthermore, psychology is of paramount importance in optimizing stress resistance in the professional environment, while positively influencing decision-making [54], communication, and the working environment of teams [55], and significantly reducing anxiety and improving physiological outcomes in patients [17]. Evidence suggests that psychological preparation can be beneficial for postoperative pain outcomes, behavioral recovery [56], negative affect, and length of stay [57]. Moreover, legal experts significantly influence the development of a sound legal and regulatory framework in anesthesiology, helping to define responsibilities and protect patient data, thereby reducing malpractice claims [58-59]. In addition, patient engagement in anesthesiology is crucial to improving the safety of care, reducing risks, and fostering effective communication between patients and the medical team. In addition, the WHO states that effective patient engagement strategies can lower the burden of harm by as much as 15% [60] while tailoring care to their preferences [61]. To reinforce this commitment, it is essential to educate patients about the risks of anesthesiology, encourage questions [62], set up accessible reporting systems, and involve them in decision-making about their treatment. This approach not only helps to improve the quality of care but also optimizes patient comfort and safety, and legal specialists ensure that anesthesiologists adhere to ethical standards, including obtaining informed consent and maintaining patient confidentiality [58]. In addition, decision-makers and politicians have an essential role to play in allocating resources [26]. They must meet the challenges facing the country by developing strategies and implementing measures that ensure effective governance [63], thus contributing to anesthesiology patient safety while complying with recommended standards [64].

Anesthesiology safety is based on close, multidisciplinary collaboration, which perfectly illustrates the spirit of the Sustainable Development Goals (SDGs). This integrated approach takes into account the economic, social, and environmental dimensions, promoting sustainable development on a global scale while responding to contemporary issues. In order to meet the complex challenges of the 21st century, the 17 SDGs of the Agenda 2030, along with their 169 targets, highlight the crucial importance of multidisciplinarity [65]. These interconnected goals require cooperation between different disciplines, fostering an enriching dialogue between exact sciences, social sciences, and political sciences [66]. This transformation is visible not only in the fields of research and education but also in public policy, demonstrating that multidisciplinarity is now essential for coordinated action in a globalized world [67]. Thus, by bringing together professionals with varied and complementary skills, whether in anesthesiology or beyond, we guarantee optimal and safe patient care, ensuring their well-being and safety while contributing to global sustainable development. In short, anesthesiology is a constantly evolving discipline, requiring integrated multidisciplinary collaboration that combines clinical expertise, technological innovation, and a thorough consideration of ethical, socio-economic, and patient engagement aspects to ensure quality care.

In parallel, contemporary research shows that interdisciplinarity is a major catalyst for innovation, increasing innovation capacity by 57% when multiple perspectives are integrated into research projects. This collaborative approach transcends traditional disciplinary boundaries, creating innovative solutions to the complex challenges of our time. What's more, it not only increases research efficiency but also transforms the way knowledge is produced, fostering the emergence of new methodologies and conceptual frameworks [68].

To realize the potential of interdisciplinarity, it is essential to develop appropriate curricula, revise assessment systems, and foster collaboration. Furthermore, scientific and technological innovation depends on overcoming disciplinary barriers to encourage a transformative and inclusive approach [69]. However, to apply these approaches on a large scale and at the level of patient safety in anesthesiology, comparative studies in a variety of fields are needed, using hybrid methodologies (quantitative and qualitative) and emerging technologies with enhanced institutional coordination, through international consortia and multi-year funding. This is essential to harmonize protocols and capitalize on knowledge. In addition, the development of decision-support tools based on open-access data is crucial to transforming research findings into effective public policies. To maximize the benefits of these approaches, comprehensive and ongoing research is needed to identify persistent challenges and develop targeted solutions.

Limitations and challenges

The success of this approach depends above all on the ability to create real synergy between all the players involved, and to put in place effective coordination mechanisms that take account of the specific features of each discipline, while placing patient safety at the heart of the priorities. However, the implementation of a multidisciplinary approach to anesthesiology in Morocco faces several major obstacles. On an organizational level, coordination between professionals remains complex, notably due to high workloads and the absence of a clear administrative framework facilitating interdisciplinary collaboration. Added to this is a significant shortage of multidisciplinary experts in areas essential to the practice of anesthesia, which limits comprehensive care and optimal risk management. In addition, cultural and psychological factors play a decisive role in resisting the adoption of innovative collaborative practices, as rigid professional hierarchies restrict open communication and hinder the emergence of informal leaders and the development of cross-disciplinary skills. In addition, resistance to change and skepticism about the effectiveness of new collaborative models slow down team buy-in. This is compounded by interpersonal conflicts that undermine team cohesion, as well as variability in patient understanding and care. On the technical front, the challenges are also significant, notably the lack of infrastructure and tools adapted to the fluid and secure sharing of information between disciplines, as well as insufficient training of non-medical professionals in safety standards specific to anesthesiology. Finally, divergent approaches and priorities between specialties further complicate the establishment of harmonious and effective collaboration.

Proposed solutions

To overcome obstacles and implement an effective multidisciplinary strategic approach to anesthesiology in Morocco, it is essential first to structure governance by creating a national committee bringing together anesthesiologists, surgeons, nurses, biomedical engineers, psychologists, lawyers, decision-makers, and members of civil society. This will ensure comprehensive and integrated coordination, overseeing actions to improve anesthesiology safety by adapting international recommendations to the Moroccan context. At the same time, an urgent review of the legal framework, involving legal experts and healthcare professionals, must clearly define the roles and responsibilities of medical and paramedical staff, while integrating international standards adapted to local constraints, and thus reinforcing confidence in the healthcare system. Thus, skills enhancement also involves joint inter-professional training programs, combining technical know-how with cross-disciplinary skills such as communication, teamwork, and stress management, to establish a culture of safety and improve the quality of care provided.

In addition, targeted investment and financial allocation for equipment modernization and the updating of rigorous protocols in line with international standards will guarantee improved technical performance. However, in order to guarantee the effectiveness of these measures, a system of regular multi-disciplinary audits needs to be implemented, which will enable us to continuously assess the safety and quality of care, identify risks, and implement appropriate corrective measures, taking into account all clinical, organizational, and human aspects. Thus, it is also crucial to develop an interactive pre-operative education program for patients using modern tools and a variety of media to foster open communication, boost patient confidence, and improve preparation.

In addition, a psychological support system is essential for the well-being of anesthesia teams, who are frequently exposed to stress and work overload, in order to prevent burnout and interpersonal tensions while optimizing collective performance. Similarly, appropriate psychological support for patients will help reduce anxiety, improve pain management, and promote better recovery. Furthermore, encouraging collaborative research between different fields represents an essential lever for strengthening innovation and adapting anesthetic practices to the Moroccan context. Moreover, promoting multidisciplinary research projects will facilitate the exchange of knowledge, the production of relevant local data, and the development of tailor-made solutions, thus consolidating the scientific and operational foundations of the discipline.

## Conclusions

In conclusion, although Morocco has made significant advances in anesthesiology, many challenges remain. Indeed, it is essential to promote integrated multidisciplinary collaboration between the various disciplines involved. This requires not only a strengthening of skills and infrastructure but also a cultural change that fosters openness, communication, and collaborative leadership, all of which are essential to the effective implementation of the proposed recommendations, such as the creation of a national multidisciplinary committee, the development of interprofessional training programs, and the revision of the legal framework, which offer a solid basis for reinforcing the safety and quality of anesthesiology care, despite the challenges associated with their application. By embarking on this path and through coordinated efforts at governmental, institutional, and community levels, Morocco can guarantee safe, accessible, quality anesthesiology care for its entire population, thus contributing to the overall improvement of its healthcare system. In addition, further collaborative research on this topic is needed to deepen and concretize this multidisciplinary approach.
